# Market assessment of fortified parboiled rice in Burkina Faso

**DOI:** 10.1371/journal.pone.0297674

**Published:** 2024-03-13

**Authors:** Alvaro Durand-Morat, Ya-Jane Wang, Imael H. N. Bassole, Lilian Nkengla-Asi, Wei Yang

**Affiliations:** 1 Department of Agricultural Economics & Agribusiness, University of Arkansas, Fayetteville, Arkansas, United States of America; 2 Department of Food Science, University of Arkansas, Fayetteville, Arkansas, United States of America; 3 Department of Biochemistry-Microbiology, Research and Training Unit/Life and Earth Sciences, University Joseph KI-ZERBO, Ouagadougou, Burkina Faso; 4 Department of Inclusive and Resilient Food Systems, Oxfam America, Washington, DC, United States of America; University of Agriculture Faisalabad, PAKISTAN

## Abstract

Micronutrient deficiency remains a daunting issue in many parts of the world. Effective interventions are needed to deal with the problem, which should consider production and consumption traditions and trends to improve their success. Parboil rice is a growing staple in Burkina Faso, where micronutrient deficiency remains high. This paper assesses the market feasibility of fortified rice through parboiling using a limited-water soaking method. Our findings suggest that consumers are willing to pay a premium for fortified rice versus conventional parboiled rice after they are informed about the importance of the problem and the potential benefits of fortified rice. A stylized cost analysis also reveals that the cost of producing fortified rice using a limited-water soaking method could exceed the premiums consumers are willing to pay, and therefore that public intervention may be needed to improve the odds of adoption by consumers. The findings have implication beyond Burkina Faso, and could guide market development in other regions where production and consumption of parboiled rice is well established.

## Introduction

Micronutrient deficiencies are widespread affecting about 2 billion people worldwide, and contribute to poor growth, intellectual impairments, perinatal complications, and increased risk of mortality [1]. Iron (Fe), zinc (Zn), and vitamin A deficiencies are prominent in countries with cereal-based diets. Iron-Deficiency Anemia (IDA) is the most prevalent and widespread nutritional disorder in the world. Zn deficiency is also prevalent and leads to adverse health consequences affecting the central nervous, gastrointestinal, immune, epidermal, reproductive, and skeletal systems [2]. Vitamin-A Deficiency (VAD) is also a major public health problem, as it is a leading cause of blindness in children, and may increase the risk of maternal mortality [3].

While the information on micronutrient deficiency in Burkina Faso is limited, findings from several studies suggest that the problem is still widespread. According to the 2019 National Nutrition Survey, the prevalence of anemia among school-aged children was 67.7%, and 83.4% among pre-school children [4]. Another study found a prevalence of anemia of 37.6% and 72.1% among women and children, respectively [5]. Globally, it is estimated that around half of the anemia cases are associated with iron deficiency [6,7]. Regarding Zn deficiency, different studies suggest a decreasing trend among the rural population in Burkina Faso, from a prevalence of 72% among children 6–31 months of age in 1999 [8], 62.7% among children 6–23 months old in 2009 [9], and 43.5% among children 6–18 months old in 2012 [10]. Another study found a prevalence of Zn deficiency of 63.7% among children, and 39.4% among women [5]. Although a decreasing trend in Zn deficiency is good news, the actual levels in Burkina Faso still remain very high. Vitamin A deficiency affects 12% of the women and 24.8% of the children in Burkina Faso [5], but its prevalence varies significantly across regions. For instance, previous studies estimated a 35% and 85% prevalence among children in the Centre-Ouest and the Centre Nord regions, respectively [11,12], a 13 to 17% prevalence among adult men and women in urban Ouagadougou [13], and 64% prevalence among women in the Centre Nord region [12].

Micronutrient deficiency is more prevalent in countries with poor dietary diversity, and positively correlated with the share of starchy staples such as cereals, roots, and tubers [14]. Rice is a major food crop in the world, and micronutrient deficiency disorders such as anemia, stunting and night blindness are widespread in most of the rice-consuming countries. According to the Food and Agriculture Organization, mass fortification is an efficient and cost-effective method to alleviate micronutrient deficiency. Being a staple crop in the world’s most densely populated regions, rice is an excellent product for delivering micronutrients through fortification [15]. Moreover, most rice produced worldwide is handled in a centralized way by millers that use paddy rice as an input in the production of milled rice, which also presents a unique opportunity for fortification interventions [16].

Rice is a growing staple in West Africa [17]. Rice consumption in Sub-Saharan Africa grew 75 in the last decade [18], the fastest growing rate worldwide, and it is expected to keep growing strongly in the coming years as population and per-capita consumption improve [19]. Rice has already become the main staple in many Sub-Saharan countries such as Mali and Senegal. In Burkina Faso, rice consumption more than double in the last decade from 420 thousand metric tons in 2010 to 850 thousand metric tons in 2020, of which roughly 1/3 is supplied domestically and 2/3 is imported [18]. Rice is a growing staple in Burkina Faso, accounting for 11.6% of the average daily caloric intake in 2020 relative to 7.8% a decade earlier [20].

There is a growing literature looking at consumer preferences for fortified foods in Africa [21–24], but to our knowledge no study to date has assessed consumer willingness to pay for fortified rice in Africa. The goal of this study is to ascertain consumer preferences for parboiled rice fortified with Fe, Zn, and vitamin A in Burkina Faso. The study is timely because rice is a growing staple in Burkina Faso, a low-income developing country with prevalence of malnutrition caused by undernourishment, and most of the rice already consumed is parboiled and, thus, similar to the fortified rice product being tested in this study. Given that fortified rice can have different appearance and culinary characteristics than non-fortified rice, it is important to assess whether consumers will accept the new fortified rice, and how much they will be willing to pay for it. Acceptance and adoption of fortified rice is crucial for the fortification efforts to help ameliorate undernourishment problems, while the willingness to pay for fortified rice has important policy implications as it will help policymakers assess the potential cost and the type of interventions, if any, that will be needed to develop a market for fortified rice.

### Rice fortification

**Fig 1:**
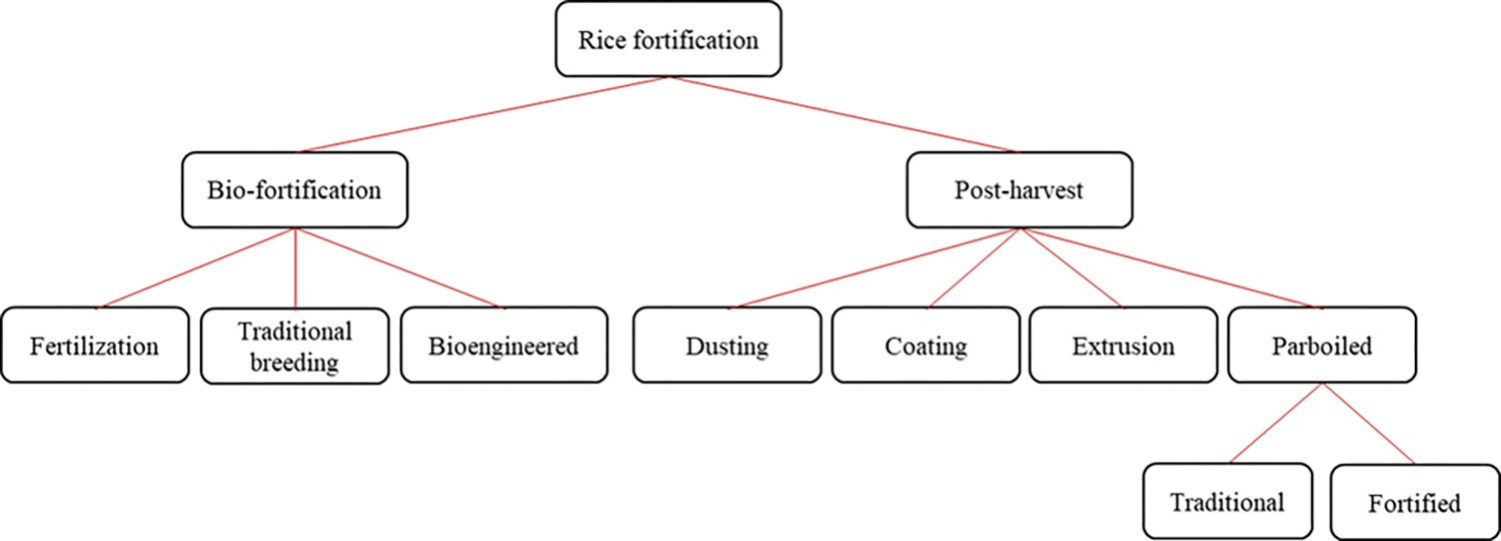
Alternative rice fortification approaches.

Fortification is the practice of deliberately increasing the content of essential micronutrients in food to improve the nutritional quality of the food supply and provide a public health benefit with minimal risks to health [25]. Rice fortification involves deliberately increasing the content of micronutrients and vitamins, such as Zn, iron, and vitamin A, to make rice more nutritious. The first trial of rice fortification was conducted in the mid-1940s in the Philippines as a way to ameliorate the impact of beriberi caused by vitamin B1 deficiency [26], and continued in the 1950s with the development of coated and extruded fortified rice blends. Rice fortification can be broadly categorized into (1) bio-fortification (genetic fortification), and (2) post-harvest fortification (Fig 1). Biofortification is a process of increasing the bioavailability of micronutrients concentrations (e.g., vitamins and minerals) in a crop through agronomic practices (e.g., foliar spray) or genetic selection (e.g., plant breeding and transgenic techniques) [27–30]. Similar to parboiled rice, micronutrients and vitamins in bio-fortified rice are embedded in all kernels and therefore less prone to losses during post-processing, cleaning, and cooking. Bio-fortified rice has the advantage of offering high levels of micronutrients even in a non-parboiled form, thus making it appealing for a broader segment of the market that already prefer and consume non-parboiled rice. Indeed, bio-fortification seeks to take advantage of the consistent daily consumption of large amounts of food staples by all family members [31].

Post-harvest fortification approaches include dusting, coating, extrusion, and parboiling techniques. Dusting consists of adding the nutrients of interest to the milled rice as a powder that sticks to the grain surface by electrostatic forces. Because of the weak bondage between the nutrient mix and the rice kernels, dusted rice is subject to significant nutrient losses when washed and/or cooked with excess water [32].

Coating involves treating rice kernels with the nutrient additive (usually powder) plus ingredients such as edible waxes and gums to improve the adherence of the mix. The coated rice kernels are then mixed with milled rice to achieve the desired level of fortification. The amount of nutrient that remains after washing and cooking the rice varies with the coating technology, but in general, coating yields better results (in terms of the amount of fortificant left available to consumers) than dusting. Some issues with coating are related to changes in color. Because the fortificant is concentrated in the surface of the kernel, coated kernels are easily distinguishable and likely discarded by consumers in the cleaning process. Moreover, if the coating film is not resistant to cooking, then the fortificant may be damaged by the exposure, and/or drained after cooking [33].

Extrusion consists of forming grain-like structures that resemble rice from a dough made of rice flour, a fortificant mix, and water. Extruded rice is then mixed with milled rice in the proportion needed to achieve the desired level of fortification. Hot extrusion uses high temperatures in the process and results in pre-cooked extruded rice with a similar appearance (e.g., transparency) to milled rice. Cold extrusion is a low-temperature process that does not use external thermal energy and results in uncooked extruded kernels that are usually more opaque and therefore easier to differentiate from regular milled rice [33]. In both hot and cold extrusion, the added nutrients are embedded in the kernel matrix, and thus largely protected from possible losses during storage, washing, and cooking. Extrusion has a high capital investment [32], which can limit its adoption in regions where fortified rice may be most needed.

Parboiling involves partial boiling of paddy (or brown) rice before milling in order to increase its nutritional value by promoting the migration of nutrients from the bran to the endosperm, to change the texture of cooked rice, and reduce the breakage in milling. Parboiling usually entails three steps, namely, soaking, steaming, and drying [34]. Adding fortificants to the soaking solution is another way to improve the nutritional value of parboiled rice [35–37]. Similar to extruded rice, the added nutrients are embedded in the endosperm, and therefore less subject to losses. Since all rice kernels are parboiled and have the same appearance, losses due to cleaning/discarding abnormal kernels are reduced. This is a viable approach for consumers that already have a habit of consuming parboiled rice.

### Fortified parboiled rice

Rice parboiling originated in India and has been used for centuries as a way to improve the physical and nutritional attributes of rice [38]. It is estimated that around 25 percent of the global rice production is parboiled every year [39]. While parboiled rice enhances the nutritional value of milled rice vis-à-vis non-parboiled rice, the nutritional improvement is constrained by the availability of nutrients in the rice bran. For instance, 100 grams of cooked long-grain brown rice, which consists of the bran and the endosperm, provides 2 percent of the daily value of iron (Fe) and no vitamin A, while 100 grams of cooked long-grain milled parboiled rice provide 1% of the daily value of iron and no vitamin A [18].

Since parboiling subjects rice to further processing before milling, different approaches have been proposed to modify the process and make parboiled rice more nutritious. Previous studies assessed the fortification of parboiled rice with (Fe) through the addition of the fortificant to the soaking solution, and found that the concentration of Fe increased between 20 to 50 times relative to that of unfortified milled rice [40]. However, the fortification process is inefficient in the sense that less than 0.10% of the fortificant added to the soaking solution actually reaches the endosperm. Such low efficiency is troublesome from an economic perspective (high production costs), and from an environmental perspective given that a large volume of fortificant is disposed together with the soaking solution and the husk [41,42]. Another studytesting the appearance and consumer acceptance of Fe-fortified rice found that the fortification with 250 mg Fe/kg of paddy rice increased the concentration of Fe up to 19.1 mg Fe/kilogram of milled rice, and had no adverse impact on consumer acceptance [37]. However, fortification with higher concentration (450 mg Fe/ kilogram paddy rice) altered the physical and culinary attributes of rice, which resulted in low acceptability. Fortification of low-amylose rice with Zn by parboiling is a cost-effective method [43], and can help fighting Zn deficiency in Bangladesh [44]. Previous studies show the feasibility of fortifying rice with Fe through parboiling using brown rice as a feedstock [45], and the benefits of using dehusked (brown) rice rather than paddy rice as a feedstock in terms of energy savings during processing and cooking [39], and also in terms of efficiency of rice fortification [36]. In an attempt to improve the water-efficiency of parboiling and reduce wastewater, a limited-water soaking method for rice fortification with calcium and Fe was found to reduced water use by 89% and the amount of solids in wastewater by 85% relative to conventional parboiling, and significantly increased the concentration of calcium and Fe in milled rice relative to conventional parboiled rice [46].

### Methodology

We used experimental auctions to assess consumer’s preferences for fortified parboiled rice in Burkina Faso. Experimental auctions are defined as a market institution for determining prices and assigning goods. Auctions have a set of rules that determine, according to the bids presented by the participating bidders, who the winner of the auctioned good is and what is the price to be paid [47]. Experimental auctions try to simulate a real market situation in which the consumer makes the decision to buy and makes the purchase, thus offering to participants real products and allowing for exchange of real money. In this way, the participant may incur real costs if he or she deviates from their equilibrium strategy, which incentivizes the participant to reveal his/her true willingness to pay (WTP) [48]. Hence, experimental auctions tend to provide more accurate WTP values than hypothetical elicitation methods [49]. We used a random *n*^*th*^ price auction as the elicitation method. With this method, the bids from each of the *m* participants are ranked in descending order, and a number *n* between 2 and *m* is selected randomly. The number *n* help determine the number of participants who receive the binding product. For example, if *n* is equal to 5, then the top 4 bidders win the auction (get the binding product) and each pay the 5^th^ highest bid. In the random *n*^*th*^ price auction, even off-margin bidders are motivated to bid their true value because it is likely that their bid is close to the market clearing price. The disadvantage of this method is that it is impossible to know the true cost of the experiment since the number of units sold in each auction increases proportionally with *n*, which is in itself unknown before each auction [50].

The research protocol was approved by the University of Arkansas’s Institutional Research Board. Participants expressed their consent to participate verbally. A team of enumerators from Ouaga University conducted 40 non-hypothetical experimental auctions between the 11^th^ and 21th of May 2022 to assess consumers’ WTP for fortified rice in Burkina Faso. We used a between-individual design to compare consumer WTP by location (urban and rural) and product type (cooked versus raw rice), and a within-individual design to account for the impact of information. We split the sample between rural and urban consumers to account for potential differences in preferences between consumers from both areas. Previous studies found significant differences in preferences for rice quality attributes between rural and urban consumers. For instance, urban consumers in Cote d’Ivoire prefer rice with very low brokens, while rural consumers choose rice with a higher broken percentage [51]. Urban consumers in Guinea prefer imported rice, while rural consumers prefer domestic parboiled rice [51]. Regarding country of origin, urban consumers in West Africa prefer imported to domestic rice [52]. Each experimental auction consisted of 10 participants, for a total of 400 participants. Half (20) of the auctions were conducted in Kienfangué (rural area), and the other half in the Zogona and Dassasgho neighborhoods in the capital city of Ouagadougou (urban area). Each auction included the following three milled rice products: (1) conventional parboiled rice, which uses paddy rice as a feedstock and is the traditional way of producing parboiled rice in Burkina Faso; (2) alternative parboiled rice, which uses brown rice as a feedstock; and (3) fortified parboiled rice, fortified with Fe, Zn, and vitamin A following the limited-water soaking method using brown rice as a feedstock as described by [46].

Furthermore, half of the auctions were for raw milled rice (no tasting) and the other half for cooked milled rice (including tasting). The physical/visual aspect of raw rice is an important search attribute that affects consumers’ purchasing decisions, while the culinary characteristics of rice are important experience attributes that can affect consumers’ repeat purchasing behavior [53]. Thus, the goal of testing both the raw and cooked rice was to ascertain if consumers’ preferences and WTP for fortified rice varies depending on whether they assess only the physical attributes via the raw milled rice, or if they also assess the culinary attributes via the cooked milled rice. The rice was cooked following the traditional method of cooking one part of rice with two parts of water. The cooking time varied from 20 to 25 minutes.

The three rice products were produced by Uneriz (National Union of Rice Parboilers of Burkina Faso). The rice parboilers at Uneriz were trained in the production of fortified rice using the limited-water soaking method, and the efficiency of the fortification process was assessed [54]. The micronutrients included in the fortification process were selected by local experts and food scientists from the University of Arkansas. The level of fortification was defined based on several factors: the Food and Drug Administration (FDA) definition of enriched rice with respect to iron, the recommended daily allowance (RDA) of these nutrients, and the physical and sensory characteristics of fortified rice. The goal was to maximize the level of micronutrients while keeping the physical and sensory characteristics of rice within reasonable levels to avoid consumer rejection due to the visual or culinary characteristics of fortified rice. The domestic rice variety TS2 was selected because of its popularity and good milling yield. With that said, the laboratory results highlight that using brown rice instead of paddy rice as a feedstock results in a lower milling yield head rice yield of 45 percent for brown rice relative to 86 percent for paddy rice). Thus, we included two controls (conventional parboiled and alternative parboiled) in an attempt to disaggregate the impact of differences in milling yields and nutritional values. The laboratory results show that the content of Zn, Fe, and vitamin A in one serving (150 grams) of uncooked milled rice of variety TS2 fortified using brown rice as a feedstock and following the limited-water soaking method amounted to 70%, 30%, and 9% of the recommended daily allowance (RDA) of adult females (Table 1).

**Table 1:** Contribution of fortified milled rice using brown rice as a feedstock and following the limited-water soaking method to the recommended daily allowance of iron (Fe), Zinc (Zn), and Vitamin A.

	Recommended Daily Allowance (mg/day)^†^	Contribution per serving (150 grams) of fortified rice (mg)	% Contribution to the RDA
Zinc	8	5.63	70.38%
Iron	18	5.48	30.44%
Vitamin A	0.7	0.07	9.32%

^†^. National Institute of Health. https://ods.od.nih.gov/HealthInformation/nutrientrecommendations.aspx.

Each auction consisted of two rounds (within-individual design). In the first round, the three rice products were presented and identified, and consumers were asked to place a bid for each of the three rice products. After the first round, participants receive the following information about fortified rice:

“Rice is a good source of calories/energy, but lacks essential nutrients and vitamins. So, if not balanced correctly, a diet heavily dependent on rice may lead to malnutrition due to insufficient minerals and vitamins. Deficiencies of Iron, zinc and vitamin A are among the leading causes of undernourishment.

Based on sound scientific information, we estimate that parboiled rice, which is the most common rice consumed in Burkina Faso, provides very little nutrients and vitamins. For example, it provides no vitamin A, around 3% of the amount of iron, and around 10% of the amount of zinc required daily by adult females.

The fortified rice used in this study has been fortified with vitamin A, iron, and zinc, and thus has higher nutritional value. For example, it provides around 9% of the vitamin A, 30% of the iron, and 70% of the zinc needed daily by adult females.”

After receiving information about fortified rice, consumers were asked to place a second bid for each rice product. Differences in WTP between the two rounds can be understood as the impact of information on consumers’ WTP.

Consumers were sampled from nearby markets following a convenience approach. Prospective participants were approached by enumerators, who briefly introduce the objectives of the research, the risks associated with participating, the expected duration, and the compensation available for completing the activity. Consumers that accepted to participate were taken to the site nearby were the auctions were performed, and signed an informed consent form acknowledging they understood the risks involved and their voluntary participation.

The auctions were performed in three steps. In step 1, each participant received an envelope containing $5 as compensation for their participation, his or her identification number, and two bidding cards. One of the main determinants of success in experimental auctions is a good and clear understanding by participants of the incentive compatibility of the auction mechanism. The survey team explained the nature of the experimental auction, including the binding nature of their bids, the importance of considering the budget constraints and own preferences when bidding for the different rice products, and the nature of the random *n*^*th*^ price auction. During the explanation, participants were encouraged to ask questions to clear any doubts about the process.

Step 2 entailed conducting the two auction rounds as described above. Finally, step 3 entailed completing the socioeconomic questionnaire and debriefing participants (S1 Table).

### Inclusivity in global research

Additional information regarding the ethical, cultural, and scientific considerations specific to inclusivity in global research is included in the (S2 Table).

### Econometric modelling

Because all bids were positive and non-censored (all bids were within a reasonable price range), we estimate a separate linear regression model to analyze the determinants of WTP for each rice product (conventional parboiled rice, alternative parboiled rice, and fortified parboiled rice) and auctioned form (raw and cooked), for a total of 6 regression models. The socioeconomic independent variables selected include 3 categories of household income, namely, (1) low-income, defined as 1 for households reporting earning less than CFA Franc 2000/month and zero otherwise, (2) middle-income, defined as 1 for households reporting earning between CFA Franc 2000 and 5000/month and zero otherwise, and (3) high-income, defined as 1 for households reporting earning more than CFA Franc 5000/month and zero otherwise. Education is defined as a binary variable equal to 1 if the participant completed any level of formal education, and zero otherwise. The share of income spent on food is represented with a binary variable equal to 1 if the share is above 50% of the monthly income, and zero otherwise. Finally, urban is a binary variable equal to 1 if the participant lives in an urban area, and zero otherwise, and household size is treated as a continuous variable. The treatment variable round (binary) represents the auction round (1 if round 2, zero otherwise), and thus is a proxy for the impact of the information treatment. Finally, we tested different interactions between socioeconomic variables and auction round to ascertain how, if any, household characteristics impact their reaction to the information treatment. For each rice product and auctioned form, the linear model is defined as:

$${WTP}_{i}=\alpha +\beta_{i}*X_{i}+\gamma_{i}*{round}_{i}+\delta_{i}*(X_{i}*{round}_{i})+\varepsilon_{i}$$ (1)

where *WTP*_*i*_ is the bid value for participant *i*; *X*_*i*_ is a vector of socioeconomic variables, *round*_*i*_ is a binary variable representing the auction round, *α*, *β*_*i*_, and *δ*_*i*_ are parameters to be estimated, and *ε*_*i*_ is the residual.

## Results

### Descriptive statistics of the sample

S3 Table shows the full experimental auction dataset. Table 2 shows the frequency of a selected group of socioeconomic variables disaggregated by location, where rural represents the surveys conducted in Kienfangué, and urban the surveys conducted in Zogona and Dassasgho. Around 80% of the participants were women, 37% were under 30 years of age, and around a third of the participants were from low-income households (earning less than CFA 2000 per month) and spent over half of their income on food. Seventy seven percent of the participants had not completed any type of formal education, compared to 51% for the population according to the Harmonized Survey on Household Living Conditions 2018–2019 [55]. Table 3 presents frequency of selected variables associated with the rice-consumption habits and preferences of participants. Around 50% of the participants report that their household consumes over 20 kg of rice per month, which divided by the average household size of 8.77 members yields over 2.3 kg per person per month or 27.4 kg per year, slightly over the 25 kg per capita per year estimated for Burkina Faso as a whole [56]. Around 2/3 of the participants report consuming parboiled rice, 20% report consuming non-parboiled rice, and 11% report not knowing which type of rice they consume. Most participants (65%) state that they closely assess the quality of the rice before buying it, while 20% of them state they trust the vendor, and 13% state they do not care about rice quality. Almost all participants report washing the rice before cooking primarily to remove impurities. Cleanliness is the main attribute of raw milled rice consumers care about, followed by color, size, and shape. The content of broken and chalk rice are the two least important attributes. For cooked rice, taste is the most important attribute, followed by swelling and texture, while aroma and stickiness are the least important. Finally, 59% (41%) of the respondents say they had (did not have) knowledge about the nutritional value of rice before the experiment, and 97% (3%) state they have (do not have) knowledge about it after the experiment. Still, when asked specific basic facts about the nutritional value of rice after the experiment, around 22% of the respondents answered it incorrectly.

**Table 2:** Frequency for some selected socioeconomic variables.

	Aggregate	Urban	Rural
Gender			
Female	322	172	150
Male	78	28	50
Age			
<30	148	74	74
31–40	120	60	60
41–50	60	31	29
>50	72	35	37
HH size	9.78	10.54	9.01
Education completed			
None	308	158	150
Elementary	70	23	47
High school	20	17	3
University	2	2	0
Income (average per HH per month)			
< CFA 2000	137	67	70
CFA 2000–3000	94	33	61
CFA 3000–5000	56	20	36
> CFA 5000	113	80	33
Income share on food			
<25%	119	50	69
26–50%	134	64	70
51–75%	82	42	40
>75%	65	44	21

**Table 3:** Frequency of selected variables associated with rice-consumption habits and preferences.

	Aggregate	Urban	Rural
Rice consumption/HH/month			
<5 kg	39	9	30
6–10 kg	48	16	32
11–15 kg	57	32	25
16–20 kg	54	29	25
>20 kg	202	114	88
Packaging			
Bagged	122	60	62
Loose	215	111	104
Both	63	29	34
Type of rice consumed			
Parboiled	274	142	132
Non-parboiled	81	36	45
Do not know	45	22	23
Type of store			
Supermarket	136	65	71
Rice wholesaler	87	57	30
Neighborhood markets	168	77	91
Other	9	1	8
Wash rice before cooking			
Always	398	200	198
Often	2	0	2
Never	0	0	0
Main reason for washing rice			
Remove abnormal kernels	52	17	35
remove impurities	315	173	142
Reduce starch in rice	9	6	3
Other	24	4	20
Ranking of characteristics of raw rice (1 = most preferred; 5 = least preferred) ^†^			
Cleanliness	1.61a	1.69a	1.53a
Color	2.08b	2.01ab	2.16b
Size	2.23b	2.07b	2.39b*
Shape	2.35bc	2.36bc	2.34b
Broken	2.60cd	2.33bc	2.88c***
Chalk	2.84d	2.53c	3.14c***
Ranking of characteristics of milled rice (1 = most preferred; 5 = least preferred) ^†^			
Taste	1.53a	1.57a	1.48a
Swelling	1.83b	1.84ab	1.82b
Texture	2.06bc	1.91b	2.20c
Color	2.26cd	2.13bc	2.40cd
Aroma	2.53de	2.33c	2.73de
Stickiness	2.72e	2.51c	2.94e
Knowledge about nutritional value of rice			
Have previous knowledge	162	86	76
Have knowledge after experiment	386	192	194
Knowledge check			
Incorrect	136	67	69
Some answers correct	175	93	82
All answers correct	88	40	48

^†^.

*, **, ***, statistically significant difference between urban and rural at 5%, 1%, and 0.1%, respectively.

ANOVA. For each location, different letters indicate statistically significant differences across attributes at the 5% level.

All bids were positive and different from zero (Table 4).

**Table 4:** Mean, minimum, and maximum bid values (CFA/kg) by location, auction form, and auction round.

Location	Auction Form	Round	Min	Mean	Max
Rural	Raw	1	200	430.16	1050
Rural	Raw	2	100	458.98	1000
Rural	Cooked	1	200	420.00	750
Rural	Cooked	2	200	447.00	1000
Urban	Raw	1	200	433.83	800
Urban	Raw	2	200	435.33	750
Urban	Cooked	1	250	420.08	600
Urban	Cooked	2	225	436.25	1250

### Willingness to pay

**Fig 2:**
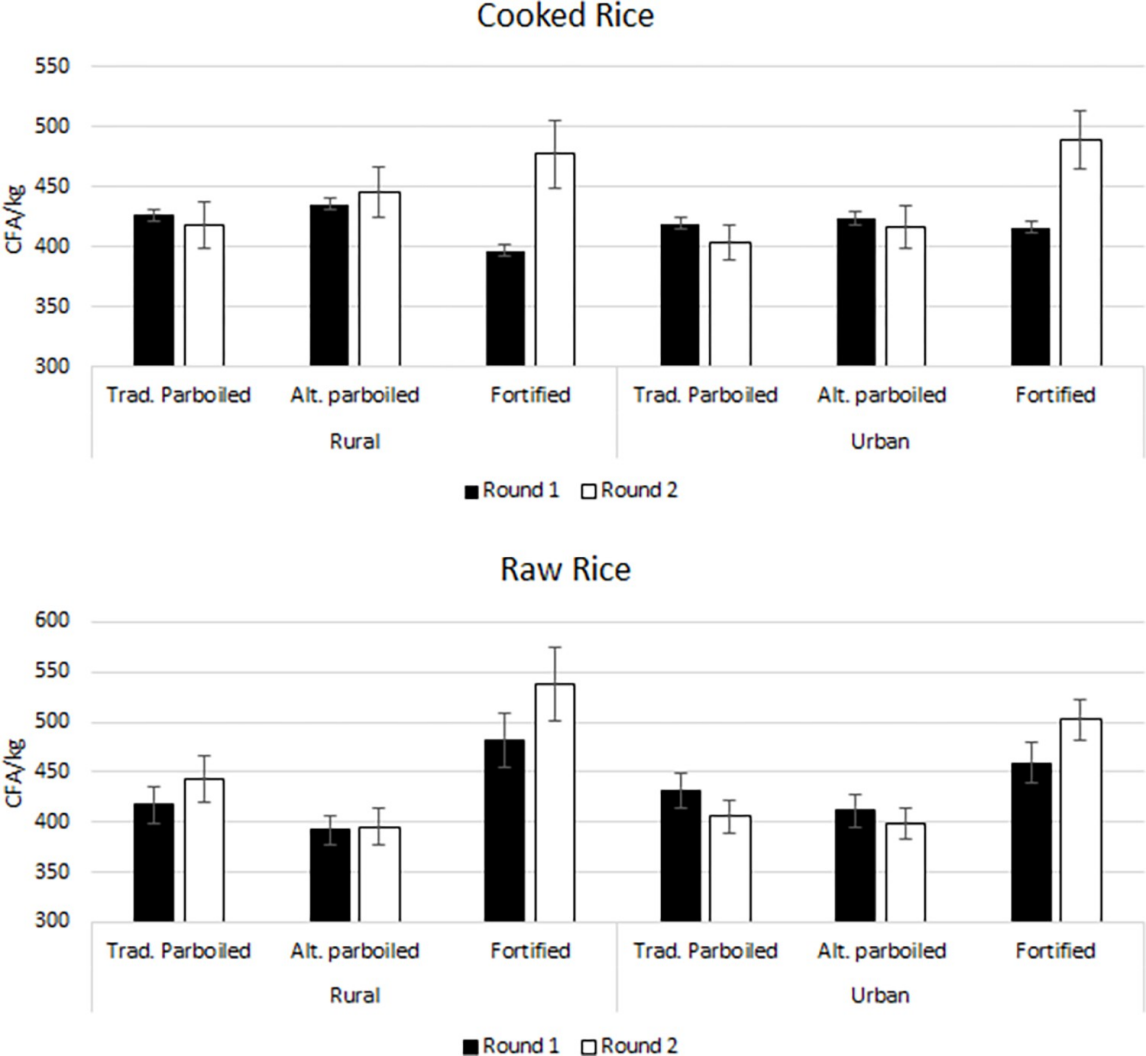
Mean and 95% confidence internal for WTP by rice product, auction form, location, and round.

Table 5 and Fig 2 show the estimated mean WTP and the results of the inference analysis to ascertain whether the differences in mean WTP are statistically significant. In aggregate, the mean WTP for 1 kg of rice varied from CFA 391.7 (US$0.63 at the average 2022 exchange rate of CFA 623.8/US$ published by the United Nations) to CFA 538.2 (US$0.86), a range deemed reasonable considering that FAO reports wholesale prices in May 2022 varying from CFA 400 to 440 per kg [57]. The results from the first round (no information about benefits of fortified rice) in the rural location suggest that consumers are WTP 15.2% (p<0.05) more for fortified rice (CFA 481.2 or US$0.77 per kg) than for traditional parboiled rice (CFA 417.6 or US$0.67 per kg) when assessing rice in its raw form, but 6.9% less (p<0.05) (CFA 397.3 or US$0.64 per kg versus CFA 426.8 or US$0.68 per kg for fortified and traditional parboiled rice, respectively) when assessing it cooked. These results may reflect that the appearance of raw fortified rice is more appealing than that of traditional parboiled rice, and consequently, consumers are willing to pay a premium for fortified rice, but that after trying the rice products, consumers may have reservations about buying fortified rice (e.g., because of some undesirable culinary aspect), and be willing to do so only at a discount. For urban consumers, the results from the first round suggest no statistical differences between consumers’ WTP for traditional parboiled and fortified rice in either the raw or cooked form, but a lower WTP for the alternative parboiled rice when tested in raw. Thus, the results suggest that, without any marketing campaign highlighting the benefits of fortified rice, consumers in urban and rural areas are willing to pay at least the same amount for fortified rice as the currently available traditional parboiled rice, but that discounts may be needed for rural households to repeat the purchase of fortified rice after trying it.

**Table 5:** Statistical analysis of differences in mean WTP^†^ in CFA per kilogram (standard deviations in parenthesis).

Rice Product	Location	Auction Form	Round 1	Round 2
Traditional parboiled rice	Rural	Raw	417.6a (93.8)	443.3a (118.4)
Alternative parboiled rice‡	Rural	Raw	391.7b (77.5)	395.5b (96.9)
Fortified parboiled rice	Rural	Raw	481.2c (137.0)	538.2c* (188.8)
Traditional parboiled rice	Rural	Cooked	426.8a (75.6)	418.3a (101.5)
Alternative parboiled rice	Rural	Cooked	436.0a (74.7)	445.3b (105.8)
Fortified parboiled rice	Rural	Cooked	397.3b (97.6)	477.5c*** (143.9)
Traditional parboiled rice	Urban	Raw	431.0a (86.6)	405.5a* (80.8)
Alternative parboiled rice	Urban	Raw	411.5b (83.2)	398.0a (78.2)
Fortified parboiled rice	Urban	Raw	459.0a (104.4)	502.5b*** (104.1)
Traditional parboiled rice	Urban	Cooked	419.8a (61.7)	403.5a (73.8)
Alternative parboiled rice	Urban	Cooked	424.0a (53.6)	416.3a* (90.1)
Fortified parboiled rice	Urban	Cooked	416.5a (79.8)	489.0b*** (123.5)

^†^. Due to the fact that the distribution of WTP is not normal, we use Wilcoxon’s rank-sum test to test differences between distributions.

‡. alternative parboiled rice is parboiled rice produced using brown rice as a feedstock.

*, **, *** indicate 5%, 1%, and 0.1% statistically significant differences between rounds for each rice product, location, and auction form.

Different letters indicate 5% statistically significant differences across rice products for each auction form in each location and in the same round.

A sensory analysis conducted as part of this research project found that urban and rural consumers perceived differences between fortified rice and the two controls (conventional and alternative parboiled) [58]. The sensory analysis of the raw samples found that fortified rice was ranked lower than the two controls. For cooked rice, the sensory analysis revealed a lower willingness to purchase fortified rice than the two control products, with significant differences in all quality attributes (general appearance, aroma, flavor intensity, overall flavor, hardness, stickiness, and texture). Counter to what we found in this study before participants received the information treatment (round 1), the sensory analysis results would suggest a lower WTP for fortified raw and cooked rice relative to the controls. One possible explanation for this counterintuitive finding is that the rice products in the sensory analysis were not identified, while they were identified as fortified, traditional parboiled, and alternative parboiled in the experimental auction. Consumers may attach a positive value to fortified rice even before receiving the information treatment (round 2).

The impact of information about the nutritional value of fortified rice can be assessed by looking at the difference in mean WTP between rounds for each product. As can be inferred from Table 5 and Fig 2, the information treatment has a positive impact on the mean WTP for fortified rice across locations and auction forms. For example, the mean WTP for fortified rice among rural consumers that auctioned cooked rice increases from CFA 397.3/kg (US$0.64/kg) to CFA 477.5/kg (US$0.77/kg) (p<0.01). This means that, as a result of the information treatment, these consumers go from being willing to pay a discount of 6.9% to a premium of 14.1% for fortified rice relative to traditional parboiled rice. The results suggest that creating a marketing campaign that highlights the benefits of fortified rice can actually increase consumers’ WTP for it above what they are WTP for other rice products, including traditional parboiled rice.

### Determinants of WTP for fortified rice

Table 6 shows the results from the OLS regression of the determinants of WTP. The Breusch-Pagan/Cook-Weisberg test revealed heteroscedasticity in one of the models, so we report and use robust standard errors for inference analysis. There is no concern of multicollinearity as the Variance Inflation Factor (VIF) values of all explanatory variables are less than 4.

**Table 6:** Results of the regression of the determinants of WTP (CFA/kg) for the three rice products auctioned.

	Traditional		Alternative		Fortified	
	Cooked	Raw	Cooked	Raw	Cooked	Raw
Middle income household	2.561 (11.565)	-10.685 (11.565)	5.456 (11.025)	-2.538 (11.025)	3.587 (15.074)	-4.015 (15.074)
High income household	18.507 (13.395)	4.16 (13.395)	31.365** (12.238)	13.546 (12.238)	9.718 (17.3)	21.68 (17.3)
Education	23.949*** (8.165)	-4.177 (8.165)	18.224** (7.966)	9.291 (7.966)	18.996 (12.287)	-21.816* (12.287)
Share income on food	-11.261 (8.424)	-3.118 (8.424)	-10.119 (8.869)	-11.888 (8.869)	-4.659 (11.829)	-20.473* (11.829)
Household size	0.681 (0.48)	1.309*** (0.48)	-0.830* (0.502)	0.389 (0.502)	-0.888 (0.677)	0.449 (0.677)
Urban	-10.129 (8.461)	-12.018 (8.461)	-16.549* (8.75)	8.03 (8.75)	21.042* (12.706)	-39.339*** (12.706)
Auction round	-12.295 (13.424)	10.421 (13.424)	9.836 (15.778)	-15.395 (15.778)	80.328*** (21.084)	23.684 (21.084)
Auction round x middle income	-0.815 (18.441)	-8.95 (18.441)	-13.799 (20.244)	27.895 (20.244)	-10.206 (27.138)	47.272* (27.138)
Auction round x high income	0.892 (20.112)	-26.046 (20.112)	-12.029 (21.652)	3.788 (21.652)	0.812 (30.579)	37.477 (30.579)
Constant	402.726*** (13.358)	425.466*** (13.358)	427.175*** (11.759)	391.359*** (11.759)	389.487*** (18.419)	508.085*** (18.419)
Observations	400	400	400	400	400	400
R2	0.046	0.019	0.052	0.022	0.115	0.069
Adjusted R2	0.024	-0.003	0.03	-0.0003	0.095	0.047
F Statistic (df = 9; 390)	2.100**	0.849	2.374**	0.988	5.633***	3.197***

*, **, *** indicate significance at 10%, 5%, and 1.0%.

Robust standard errors in parenthesis.

Overall, only four of the six models are significant, and those that are significant have a very low goodness of fit. The overall fitness of the models reveals that the set of independent variables used offers a very limited explanation of the WTP for the three different rice products and locations. This finding can be seen as positive in the sense that it supports the creation of general marketing and policy approach for the population at large, instead of tailored strategies for different segments. This could lead to more cost-effective approaches to advance the adoption of fortified rice.

There are some differences in the determinants of WTP worth discussing. Looking at fortified rice, the results suggest that WTP is significantly (p < 0.10) and negatively affected by education when auctioned in raw form, which may impact first-time purchases, but it has no impact when auctioned as cooked rice, which is important as this may determine the repeated-purchasing behavior. The share of income spent on food has a significant (p < 0.10) negative impact on WTP in raw form, and no significant impact on WTP for the cooked rice. Urban households have a significant (p < 0.01) negative impact on WTP for raw fortified rice, but significantly (p < 0.10) positive impact on the WTP for cooked fortified rice.

The auction round, which is a proxy for the value of information, is significant (p < 0.01) and positive for fortified cooked rice, but insignificant for raw fortified rice. Looking at the interaction terms, the results show that the WTP of middle-income households increases significantly (p < 0.10) as a result of the information treatment, while income level does affect the impact of the information treatment on the WTP for fortified cooked rice. The auction round and its interactions with income do not have any significant impact on the WTP for the other two rice products (traditional and alternative parboiled rice).

## Discussion and policy implications

Most of the domestic rice in Burkina Faso is parboiled using paddy rice as a feedstock, which corresponds to the conventional parboiled rice used in this study as one of the control products. Thus, when addressing the question of whether there is market potential for the fortified parboiled rice analyzed in this study, we focus the analysis on the results for traditional parboiled rice and fortified rice.

As discussed in the results section, without information about the benefits of fortified rice, rural consumers stated they are willing to pay a premium of 15.2 percent for raw fortified rice, but a discount of 6.9 percent after trying it (cooked). On the other hand, urban consumers stated the same WTP for both rice products in raw and cooked form. Urban and rural consumers alike stated they would be WTP a premium for fortified rice after receiving information about the benefits of fortified rice. Thus, the first recommendation for policymakers and industry stakeholders is that any attempt to market fortified rice produced using the limited-water soaking method must be accompanied by a clear marketing campaign highlighting the nutritional benefits of fortified rice and the prevalence of undernourishment in Burkina Faso. The economic benefit of a marketing campaign can be estimated as the difference in WTP before and after the information treatment as reported in Table 5. We estimate the benefits of a marketing campaign to vary between US$69.7 to US$128.6 per metric ton of fortified milled rice, or between US$1.74 and US$3.22 per person considering that a metric ton of rice is enough to feed 40 people per year in Burkina Faso at the per-capita consumption rate of 25 kg per year [56]. These benefit estimates can be used by the industry and policymakers to assess, for instance, the cost-benefit ratio of creating a marketing campaign under different assumptions of effectiveness (e.g., share of consumers reached by the marketing campaign that start consuming fortified rice). The regression results support the idea of developing a broad marketing and policy strategy since the models have very low explanatory power. Broad marketing and policy strategies have the appeal of being easier and likely less costly to implement than targeted approaches, which could lead to a higher cost-effectiveness and likelihood of success in the adoption of fortified rice. However, despite the lack of significant explanatory power of the selected socioeconomic variables, the problem of undernourishment in Burkina Faso is more severe among low-income households [59], and therefore promoters must ensure they reach the most vulnerable segments where the problem is more acute.

An important question that remains is whether the stated price premiums consumers are willing to pay is enough to cover the cost of fortifying rice. While the limited-water soaking method results in less water and energy use relative to conventional parboiling [46], the method of fortification assessed in this study could be expected to cost more than producing conventional parboiled rice, primarily because it requires the use of fortificants, vacuum plastic bags, and also more labor and an extra round of drying. In Burkina Faso, conventional sun drying on tarpaulin is the most common drying method used due to its low investment costs and easy handling [60], which implies that an extra drying round should not be costly. However, it is difficult to assess the cost of one extra round of drying. Also, vacuum plastic bags can be recycled and used multiple times, which should result in a marginal cost per metric ton. Thus, we expect the highest cost would come from fortificants and labor.

Table 7 below shows a simple estimation of the cost of production of fortified rice using the limited-water soaking method. At the concentrations of fortificants in the soaking solution recommended by [54], the total cost of fortificants is US$69.5 per metric ton of fortified rice. Regarding labor, anecdotal references from the Uneriz parboilers themselves suggest that, without counting soaking time (which is the same with both methods), parboiling 100 kg of rice using the traditional method takes around 6 hours of labor, while parboiling using the limited-water soaking method takes around 10.5 hours of labor, that is, an increase in labor use of 75%, or 45 more hours of labor per metric ton. Assuming an average wage of US$3.2/hour [61], we estimate the extra labor cost to be US$144 per metric ton. Hence, considering the estimated cost of fortificants (US$69.5/mt rice) and labor (US$144/mt rice), the extra cost of fortification amounts to US$213.5 per metric ton of fortified rice.

**Table 7:** Cost (US$/metric ton of rice) of producing fortified parboiled rice using the limited-water soaking method, and its relationship with the estimated price premiums (US$/metric ton of rice).

Cost Items^†^ ^‡^	Cost	Location and auction form	Premium	Cost–Premium	Premium/Cost
*Ferrous sulfate*	*20*.*0*	Rural Cooked	95	118.5	44%
*Zinc sulfate*	*7*.*5*	Rural Raw	152.2	61.3	71%
*Dry Vitamin A*	*42*.*0*	Urban Cooked	85.5	128.0	40%
*Labor cost*	*144*.*0*	Urban Raw	155.5	58.0	73%
Total	213.5				

^†^. Ferrous sulfate (FeSO4.7H2O): Molecular weight 277.9; Fe content 20.1%. Zinc sulfate monohydrate (ZnSO4.H2O): Molecular weight 179.5; Zn content 36.4%. Vitamin A (C20H30O): Nutrient content 100%.

^‡^. Prices for the fortificants were collected in September 2022.

The nominal value of the stated price premiums for fortified rice after receiving information about the benefits of fortified rice amounts to US$147.1/mt for raw rice and US$91.8/mt for cooked rice among rural consumers, respectively, and US$150.4/mt for raw rice and US$132.6/mt for cooked rice among urban consumers, respectively. Thus, the stated premiums are enough to cover the cost of fortificants, but not sufficient to cover the total cost of production of fortified rice using the limited-water soaking method, and that therefore external support will be needed to increase the likelihood of adoption.

The support needed, estimated as the difference between the premiums and the additional cost of production, varies between US$58 and US$128 per metric ton. To put this value in perspective, a metric ton of rice is enough to feed 40 people per year in Burkina Faso at the per-capita consumption rate of 25 kg per year [56], which amounts to between US$1.45 and US$3.20 per person per year in external support. These costs of external support can be used to estimate the total cost of external support needed to implement a rice fortification program using the limited-water soaking method. Thus, a program targeting 100% of the consumers of parboiled rice in Burkina Faso (68.5 percent of the population according to Table 3, which amounts to 15.5 million people) would require between US$22.5 and US$ 49.6 million.

While estimating the energy savings resulting from the use of the limited-water soaking method relative to conventional parboiling is beyond the scope of this study, it is important to mention its potential relevance in the context of improving energy use efficiency and sustainability. The process of parboiling as practiced in rural areas is time-consuming, laborious and energy intensive [62]. Wood is the main source of fuel used in parboiling in Sub-Saharan Africa, which usually entails long transportation distances to processing centers, and its burning contributes to lower air quality and respiratory illness [63].

## Conclusion

Rice is a great vehicle in the fight against undernourishment in Burkina Faso because of its growing importance as a staple food. Moreover, fortification programs based on the parboiling process are likely to be more easily accepted by producers given the long tradition of parboiling rice in Burkina Faso. However, the feasibility of a new product depends on whether or not consumers will accept it and adopt it.

Fortified rice using the limited-water soaking method has the potential of helping fight micronutrient deficiencies in Burkina Faso. The results from the experimental auctions suggest that consumers are WTP the same price for fortified rice than for traditional parboiled rice, except for rural consumers that assessed the cooked rice. Overall, these findings are troublesome in the sense that the increased cost of producing fortified rice cannot be passed to consumers via price premiums, and would rather need to be covered by other means, which could include government support (e.g., production subsidy). The results also highlight the significant and positive impact of information on the mean WTP for fortified rice. After receiving information about the benefits of fortified rice, consumers’ WTP increased significantly, leading to sizable price premiums over traditional parboiled rice. These findings are encouraging and speak of the importance of developing and deploying effective information campaigns, which can result in consumers bearing at least part and potentially all the increase in production cost through rice price premiums.

The adoption of the limited-water soaking method for rice fortification could increase production costs beyond the value of the price premiums. While production costs may decrease with time as rice parboilers gain experience with the new method, our findings indicate that external financial support may be needed to improve the marketability of fortified rice. Whether this approach is cost-effective compared to other types of interventions used in Burkina Faso to ameliorate undernourishment is something policymakers and health officials should evaluate before committing to support the production of fortified rice using the method assessed in this study.

The findings of this study have implications for other countries and regions where consumption of parboiled rice is well established, such as in rural areas in Benin, Guinea, and northern Ghana where domestic parboiled rice dominates, in urban settings in southern Nigeria where imported parboiled rice dominates, and in general among consumers in Liberia and Niger where low-quality domestic parboiled rice is commonly consumed [51]. Consumption of parboiled rice is also prominent in South Asia given that over 50% of the rice produced in South Asia is parboiled [64]. A more recent study revealed that farming households in Bangladesh prefer parboiled rice [65]. A potential advantage of trying the limited-water soaking method in other regions with commercial, large-scale parboiling plants is the potential cost savings due to scale, which can improve the economic feasibility of fortified rice.

## Supporting information

S1 Table Socioeconomic questionnaire. (PDF)

S2 Table Inclusivity in global research. (DOCX)

S3 Table Experimental auction dataset. (XLSX)
