# Supplementary material for: Market assessment of fortified parboiled rice in Burkina Faso
Source: PLoS One. 2024 Mar 13;19(3):e0297674. doi: 10.1371/journal.pone.0297674 (PMC10936829; doi:10.1371/journal.pone.0297674)
Supplement: S1 Table — (PDF) [file pone.0297674.s001.pdf]

Socioeconomic Questionnaire

1. Gender
   1. Male
   2. Female
   3. Other

2. Age
   1. 30 years or less
   2. 31-40 years
   3. 41-50 years
   4. 51 years or more

3. How many people live and/or consistently share meals in your household? _____

4. How many children (under 5 years of age) live in your household? _____

5. Education degree completed by respondent
   1. Elementary school incomplete
   2. Elementary school complete
   3. Secondary school incomplete
   4. Secondary school complete
   5. University or post-secondary school incomplete
   6. University or post-secondary school complete
   7. Other____
   8. None

6. Average monthly household income (counting all sources of income from all household members)
   1. Less than CFA Franc 2000
   2. Between CFA Franc 2000 - 3000
   3. Between CFA Franc 3000 - 5000
   4. More than CFA Franc 5000

7. What share of the household income is spent on food?
   1. 25% or less
   2. 26%- 50%
   3. 51% - 75%
   4. 75% or more

8. How much rice is consumed in your household every month (estimated average)?
   1. 5 kg or less
   2. 6 Kg – 10 Kg
   3. 11 Kg – 15 Kg
   4. 16 Kg – 20 Kg
   5. 21 Kg or more

9. How often do you eat rice?
    1. Every day, one time a day
    2. Every day, more than one time a day
    3. Not every day, but at least 4 days a week
    4. Not every day, and less than 4 days a week

10. How much did you pay last time you purchased rice (approximate, CFA Franc/kg)? _________

11. What is the primary way you buy rice?
    1. Loose
    2. Bagged/ Packaged
    3. Both
    4. Other _______________________________________

12. What type of rice does your household primarily consume?
    1. Parboiled
    2. Non-parboiled
    3. I do not know

13. Where do you usually buy your rice?
    1. Supermarkets
    2. Rice wholesaler-retailer
    3. Neighborhood markets
    4. Other _______________________________________

14. How do you assess the quality of the rice you buy?
    1. I searched around, look and smell the rice to ascertain the quality before I buy.
    2. I trust the vendor and always buy the same rice from the same vendor without hesitation.
    3. I do not care much about the quality of the rice I buy
    4. Other _______________________________________

15. Do you wash and clean the rice before cooking?
    1. Always
    2. Often
    3. Rarely
    4. Never

16. If you wash and clean the rice before cooking (answer a, b, or c in (15)), what is the main reason for that?
    1. To remove abnormal kernels
    2. To remove impurities
    3. To reduce starch in rice
    4. Other _______________________________________

17. Rank the following characteristics of uncooked rice based on your own preferences (1 = most preferred, 5= least preferred). You can rank more than one characteristic at the same level, for instance, if you value cleanliness and color/whiteness the most, then put 1 for both)
    a. _____Cleanliness – whether uncooked rice is clean
    b. _____Color/whiteness – whether uncooked rice is white, yellow, or have some off color
    c. _____Broken rice – amount of broken rice, defined as a kernel that is less than 75% of the length of a whole kernel, in the uncooked rice
    d. _____Chalk (opaque) rice – number of opaque/chalk kernels in the uncooked rice
    e. _____Size – whether kernels are long or short
    f. _____Shape – rice that looks slender, medium, or bold/coarse
    g. _____Other __________________________

18. Rank the following characteristics of cook rice based on your own preferences (1 = most preferred, 5= least preferred). You can rank more than one characteristic at the same level, for instance, if you value aroma and texture the most, then put 1 for both
    a. _____Aroma – whether cooked rice has a fragrance or not
    b. _____Texture – whether cooked rice have a soft, hard, or chewy consistency
    c. _____Stickiness – whether kernels stick together or remain loose when cooked
    d. _____Swelling – whether kernels elongate/grow when cooked
    e. _____Color/whiteness – whether cooked rice is white, yellow, or have some off color
    f. _____Taste
    g. _____Other __________________________

19. Before this experience, were you aware of the nutritional benefits of rice?
    1. Yes
    2. No

20. After this experience, do you think you understand better the nutritional benefits of rice?
    1. Yes
    2. No

21. Mark the statements below that you think are correct.
    1. Parboiled rice has a higher nutritional value than non-parboiled rice
    2. A rice-based diet provides enough minerals and vitamins for a healthy lifestyle
    3. Rice is a good source of calories but a poor source of minerals and vitamins
    4. Fortification is a way to improve the nutritional benefits of rice

22. Have you changed your purchasing habits based on the recent COVID-19 pandemic?
    1. Yes
    2. No

23. If you answer "yes" in (22), why? (you can mark more than one option)
    a. Because of the changes in food prices, including rice
    b. Because of a change in income
    c. Because of limitations to access food due to the restrictions imposed by the pandemic
    d. Other____

24. If you answer “yes” to (22), have you purchased more or less rice than usual?
    a. More (around _____% more)
    b. Less (around _____% less)

25. Based on your experience, during the COVID-19 Pandemic the price of rice has:
    a. Increased
    b. Decreased
    c. Stay the same

Comments

